# Understanding Photo(electro)catalysts for Energy Conversion via Operando Functional Imaging

**DOI:** 10.1021/cbmi.3c00074

**Published:** 2023-08-30

**Authors:** Weidong Zhang, Shurui Chen, Kai Shen, Jing Zhu, Yong Liu, Xianwen Mao

**Affiliations:** † Department of Materials Science and Engineering, National University of Singapore, Singapore 117575, Singapore; ‡ Department of Chemical and Biomolecular Engineering, National University of Singapore, Singapore 117575, Singapore; § Institute of Functional Intelligent Materials, National University of Singapore, Singapore 117575, Singapore; ∥ Center for Advanced 2D Materials, National University of Singapore, Singapore 117575, Singapore

**Keywords:** operando functional imaging, photo(electro)catalyst, energy conversion, charge carriers, single particle, surface photovoltage, photoluminescence, single-molecule fluorescence microscopy

## Abstract

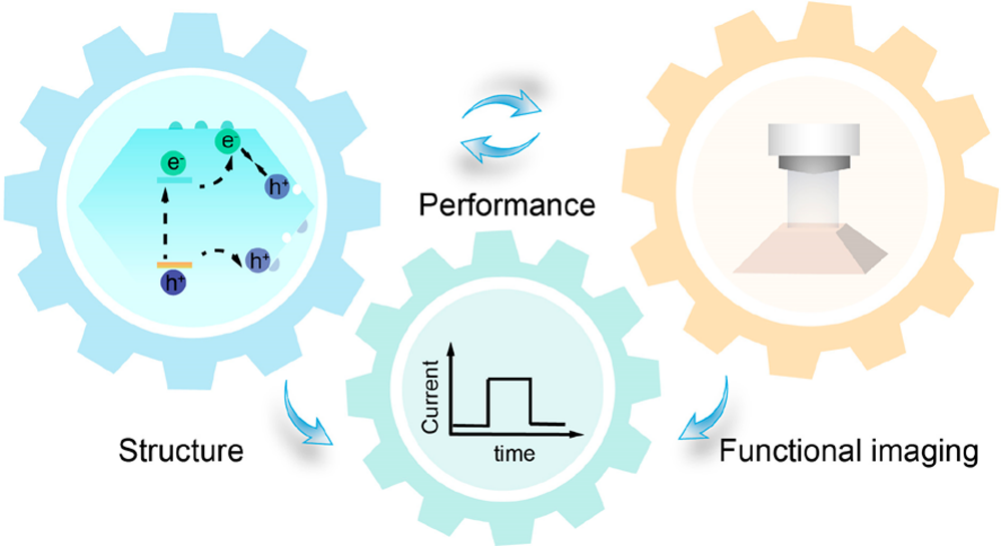

Understanding the fundamental processes of charge carrier generation, separation, transport, and reactivity on the surface of semiconductors is crucial for the rational design of high-performance photo(electro)catalysts for various energy conversion applications. Nevertheless, the ubiquitous and intricate heterogeneity exhibited by individual catalysts poses a significant obstacle to achieving a comprehensive understanding of the structure–function relationships using bulk-level, ensemble-averaged characterizations. This review highlights the emerging operando imaging techniques capable of providing local functional information, such as surface photovoltage, photoluminescence, charge carrier reaction rate, and photocurrent, at the single particle to subparticle level. By establishing correlations between the acquired local functional information and the specific structural characteristics of the catalyst, a quantitative, holistic understanding of the structure–function relationship in photo(electro)catalysts can be achieved. This understanding can serve as a foundation for guiding the design of photocatalysts with enhanced energy conversion performance.

## 1 Introduction

Sunlight, as the most abundant natural resource on Earth, represents a highly desirable and sustainable energy source with the potential to replace fossil fuels.^1,2^ The amount of solar energy reaching the Earth’s surface is approximately 1.3 × 10^5^ terawatts (TW), which is nearly 10^3^ times the current global energy consumption.^3^ Photoelectrochemical (PEC) and photocatalytic (PC) processes utilizing semiconductors with light-harvesting and catalytic capabilities play a crucial role in processes converting solar energy into value-added chemicals, such as water splitting, H_2_O_2_ production, and CO_2_ reduction.^4−6^

Metal oxide semiconductors with suitable band gaps and chemical stabilities have garnered significant research interest since the discovery of TiO_2_-mediated PEC water splitting five decades ago.^7^ However, despite years of advancements, solar-driven photocatalysis has not yet emerged as a cost-competitive technology for practical applications due to its poor solar-to-energy conversion efficiency.^8^ For instance, in most reported photocatalytic systems, the solar-to-hydrogen (STH) efficiency of water splitting remains below 3%, leading a high overall cost of hydrogen production.^9,10^ Plasmonic nanoparticles have recently attracted considerable interest as promising photocatalysts, primarily driven by the mechanism of localized surface plasmon resonances.^11,12^ When illuminated, these nanoparticles exhibit the generation of energetic charge carriers, elevated surface temperatures, and intensified electromagnetic fields, all of which play pivotal roles in the photocatalytic process.^13,14^ Nonetheless, the intricate interplay of these plasmonic mechanisms poses challenges to the practical implementation of plasmonic photocatalytic systems.^15^ Gaining a deeper understanding of the mechanisms underlying solar-driven photocatalysis processes is essential for the development of highly efficient photocatalysts.^16^

**Figure 1:**
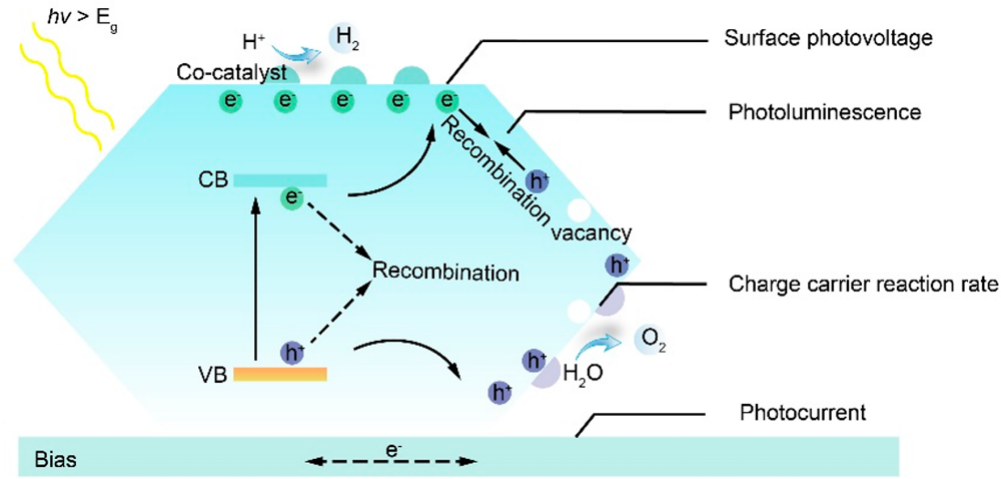
Fundamental processes involved in typical photo(electro)catalysis and key functional parameters governing the overall performance under light excitation.

As shown in Figure 1, photocatalysis (or photoelectrochemical catalysis) involves several key fundamental processes: (1) *Charge generation*: semiconductors with optical bandgaps in the visible or near-ultraviolet range can absorb solar irradiation, promoting the excitation of electrons from the valence band to the conduction band. The positions of the valence and conduction bands determine the redox capability of the generated holes and electrons, which enables selective catalytic reactions.^17^ (2) *Charge separation*: semiconductors possess unique electronic properties that lead to the formation of charge carrier depletion regions extending from the bulk to the surface. This phenomenon facilitates charge separation by inducing band bending toward the surface. The degree of band bending indicates the ability of minority charge carriers to reach the surface, and it can be assessed through surface potential measurements. Illumination induces a change in surface potential, leading to the occurrence of surface photovoltage (SPV) phenomena. SPV serves as a promising technique for monitoring both charge separation efficiency and the direction of band bending.^18^ (3) *Charge transport:* before reaching the surface and engaging in reactions, a significant fraction of photogenerated electrons and holes recombine with each other, resulting in the release of heat or photons. This recombination process contributes to the poor efficiency of converting absorbed photons into current. Radiative recombination, which can occur through band-to-band relaxations or the recombination of trapped charge carriers at defect sites within the bulk or on the surface of the semiconductor, can be discerned by observing the emitted light at different wavelengths.^19^ (4) *Interfacial reaction*: charge carrier-induced surface reactions constitute the subsequent stage after charge separation and transport, involving the transfer of charge carriers toward reactants or intermediate species at the interfaces that bridge the semiconductor and liquid phases.^20^ Importantly, the charge transfer barrier depends on the adsorption behavior of reaction intermediates. An optimal adsorption strength for these intermediates significantly enhances the activity and selectivity of related reactions (e.g., water splitting).^21^ Control over the adsorption strength of intermediates is key to obtaining better catalytic properties. Moreover, another extensively utilized approach to enhance performance involves the incorporation of cocatalysts, a widely adopted strategy for refining semiconductor surfaces and thereby amplifying their catalytic efficacy.^22,23^ Notably, for photocatalysis, a measurable parameter that assesses overall semiconductor performance is the photocurrent, serving as an indicator mirroring rates of oxidation and reduction half-reactions.^24^ Applying a positive or negative bias to individual photoelectrochemical cells proves instrumental in propelling the desired half-reactions, effectively steering the photocatalytic processes toward desired outcomes.

Improving the charge separation efficiency and interfacial activity of photogenerated charge carriers is the primary objective in the field of photo(electro)catalysis.^12^ Although no photocatalysts have achieved the target goal of achieving an STH efficiency greater than 10%, significant progress has demonstrated that the photocatalytic efficiency can be enhanced through precise chemistry, such as heterojunctions^25^ and defect engineering,^26^ as well as nanostructure designs including hierarchical architecture control,^27^ crystal-facet engineering,^28^ and cocatalyst loading.^29^ Anisotropic semiconductors with heterogeneous facets, such as BiVO_4_ and BiOBr particles,^30,31^ have been found to facilitate interfacet charge separation, enabling the accumulation of light-generated holes and electrons on separate facets. The introduction of reduction and oxidation cocatalysts on specific facets has proven to be an effective approach for further improving catalytic kinetics. By selectively depositing reduction cocatalysts (such as Au and Pt) and oxidation cocatalysts (transition metal oxides and hydroxides) on different semiconductor facets, the half-reactions of hydrogen evolution and oxygen evolution in water splitting can occur on spatially separated facets.^32^ It is worth noting that these strategies often necessitate precise control of nanoscale structures and local chemistry, resulting in increased structural complexity and heterogeneity of the particulate semiconductors.

While ensemble-level electrochemical tests and characterizations provide a convenient means of evaluating the overall performance of photoelectrodes, they may not fully reveal the heterogeneous reactivity which can only be probed by single-particle-level techniques.^33,34^ Therefore, to advance catalyst designs, it is important to obtain a deep understanding of the correlation between local structural information and functional performance, especially at the single or subparticle level.^35−37^ Additionally, in addition to the static structure–reactivity heterogeneity among single/subparticles, the electrochemical active sites and local microenvironments of individual photocatalysts undergo dynamic changes during operation under working conditions such as light illumination and electrochemical control in a reaction electrolyte.^38^ Therefore, it is crucial to employ operando characterization techniques that provide high spatiotemporal resolution to comprehensively understand the dynamic evolution of the structure–reactivity relationship.

In this review, our focus is on the latest advancements in emerging operando imaging techniques with high spatial and temporal resolutions to provide local functional information about semiconductor photocatalysts. These techniques encompass surface photovoltage, photoluminescence, charge carrier reaction rate, and photocurrent measurements at single particle level. The primary objective of these operando functional imaging techniques is to address the challenges posed by the inherent heterogeneity of individual particles and gain insights into the local structural–functional relationships in photocatalytic materials. The high temporal resolution provided by these techniques enables the observation of dynamic changes in functional properties, including charge carrier dynamics and reaction kinetics, throughout the reaction process. By correlating the local functional information acquired through these techniques with the catalyst’s local structural properties, key features and mechanisms that govern photocatalytic performance can be identified. Most importantly, the insights obtained from operando functional imaging techniques can guide the rational design of photocatalytic materials by offering a deeper understanding of the structure–function relationships.

## 2 Operando Functional Imaging To Monitor the Local Structure–Reactivity Relationship

### 2.1 Facet-Dependent Surface Photovoltage

**Figure 2:**
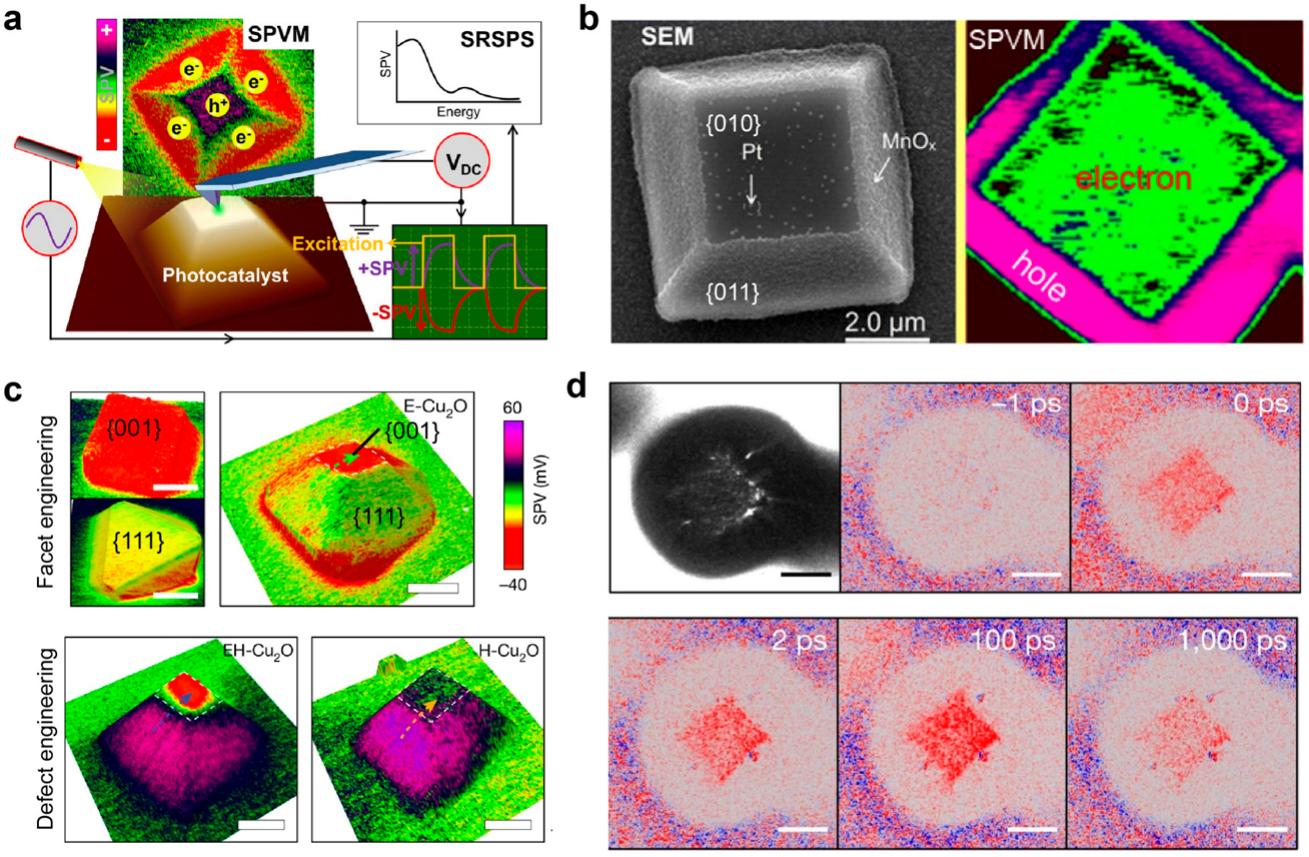
Schematic and application of surface photovoltage microscopy. (a) Illustration of the experimental realization of SPVM based on Kelvin probe force microscopy and spatially resolved surface photovoltage spectroscopy. Reprinted with permission from ref (18). Copyright 2022, Wiley-VCH GmbH. (b) Scanning electron microscopy (SEM) and SPVM images of a single BiVO_4_ particle that facet-selectively loads MnO_*x*_ and Pt dual cocatalysts. Reprinted with permission from ref (32). Copyright 2017, American Chemical Society. (c) SPVM images of facet-engineered Cu_2_O particles of cubic and octahedral morphologies (top left), defect-engineered truncated octahedral Cu_2_O particles without (top right), with moderate density of (bottom left), with extreme incorporation of hydrogen-compensated copper vacancy defects. Scale bars: 2 μm. Reprinted with permission from ref (42). Copyright 2022, Springer Nature. (d) Photoemission electron microscopy image and time-resolved photoemission electron microscopy image series of truncated octahedral Cu_2_O particle that is moderately hydrogen-compensated copper vacancy defect incorporated. Scale bars: 2 μm. Reprinted with permission from ref (42). Copyright 2022, Springer Nature.

Surface photovoltage microscopy (SPVM) combines Kelvin probe force microscopy (KPFM) with illumination tools such as xenon lamps, LEDs, and lasers to illuminate photocatalysts. The catalysts are excited by absorbing photons, resulting in super band excitation near the surface, which corresponds to variations in the surface potential measured by KPFM.^39,40^ SPVM enables the spatially resolved measurement of surface photovoltage by subtracting the contact potential difference (CPD) measured in the dark state from the CPD measured under illumination (Figure 2a). In SPVM, a positive surface photovoltage indicates the presence of holes, while a negative value indicates the presence of electrons. The magnitude of the surface photovoltage is directly related to the density of separated photogenerated charges and their separation distance, providing valuable insights into the distribution and migration of photogenerated charges.^16,41^

SPVM has been successfully applied to map the distribution of spatially separated photogenerated charge carriers on the surface of single crystals of the BiVO_4_ photocatalyst and to study the function of loaded cocatalysts.^32^ Specifically, MnO_*x*_ and Pt cocatalysts were selectively loaded onto the {011} and {010} facets, as shown in Figure 2b. Scanning electron microscopy (SEM) images confirmed the deposition of MnO_*x*_ nanoparticles, which are known for their role in the oxygen evolution reaction, on the {011} facet, while Pt was deposited on the {010} facet, promoting the hydrogen evolution reaction. SPVM was employed to investigate the influence of these dual cocatalysts on the process of photogenerated charge separation. The resulting potential distribution map revealed distinct differences between the two facets, with a strong positive SPV value observed on the lateral {011} facet and a negative SPV value on the basal {010} facet. This observation indicates that photogenerated holes are selectively separated on the {011} facet, while photogenerated electrons accumulate on the {010} facet. The facet-selective separation of charges is attributed to the presence of the cocatalysts, which enhance the built-in electric field of the BiVO_4_ particles, thereby driving the separation and migration of photogenerated charges. In addition to their well-known functions in interfacial charge transfer and thermal catalysis, the facet-selectively loaded cocatalysts alter the direction of the built-in electric field beneath the unloaded surface of BiVO_4_ by aligning the electric field vectors. This alignment significantly strengthens the local electric fields, leading to an enhancement of up to 80 times through the synergistic effect of electric field direction alignment.

In addition to investigating the role of cocatalysts, SPVM has emerged as a powerful tool for studying the distribution of near-stable-state separated photogenerated charges on photocatalysts at the single-particle level and exploring the influence of asymmetric facets and defect engineering, as illustrated in Figure 2c.^42^ By controlling the surface area ratio of {001} facets to {111} facets in Cu_2_O particles, which serve as a promising system for understanding photogenerated charge separation, SPVM analysis reveals varying degrees of photogenerated charge accumulation based on the facet ratio of the Cu_2_O particles. Cubic particles with a high {001} to {111} facet ratio exhibit a strong negative surface photovoltage on the {001} facets, while octahedral particles with a low {001} to {111} facet ratio demonstrate a weaker negative SPV on the {111} facet. Importantly, it is observed that photogenerated electrons preferentially accumulate on the {001} facet, attributed to the higher density of copper vacancies on this facet, resulting in a more pronounced p-type character. The disparity in copper vacancy distribution generates interfacet built-in electric fields that act as driving forces for photogenerated charge transfer. SPVM confirms that by tuning the facet ratio, specifically by achieving a truncated octahedral morphology, photogenerated electrons accumulate preferentially on {001} facets rather than {111} facets, resulting in a more negative SPV on {001} facets than on {111} facets.

To comprehensively understand the photogenerated charge transfer process, it is advantageous for electrons and holes to transfer to different facets, spatially separating them on the surface of photocatalyst particles. To achieve this, spatially adjustable anisotropic defect engineering is employed by introducing hydrogen-compensated copper vacancy defects, which act as hole extractors, exclusively to the facets of Cu_2_O particles. SPVM characterization demonstrates that after introducing hydrogen-compensated copper vacancy defects exclusively to {111} facets, the accumulation of photogenerated electrons and holes exhibits facet-dependent spatial selectivity. Specifically, electrons preferentially transfer to the {001} facet, while holes accumulate on the modified {111} facets. These Cu_2_O particles that exhibit both electron and hole accumulation are denoted as EH-Cu_2_O. Further incorporation of hydrogen-compensated copper vacancy defects into truncated octahedral Cu_2_O particles, resulting in the enrichment of both {001} and {111} facets with the defects, leads to the disappearance of negative SPV values. This suggests that only holes accumulate on the surface of the photocatalyst particles, categorized as H-Cu_2_O. The transient evolution of SPVM results provides insights into the impact of facet and anisotropic defect engineering on the charge transfer dynamics of photocatalysts.

For a more comprehensive understanding of the charge transfer process, particularly to investigate the ultrafast interfacet electron migration process occurring within a single EH-Cu_2_O particle upon photon excitation, time-resolved photoemission microscopy (TR-PEEM) is employed (Figure 2d).^42^ TR-PEEM enables the visualization of charge transfer dynamics of photocatalysts on the femtosecond to nanosecond time scale at the single-particle level. By capturing a sequence of images at different pump–probe delays, TR-PEEM provides snapshots of the ultrafast photogenerated electron transportation driven by interfacet electric fields. The series of images obtained through TR-PEEM reveal that upon pulsed photon excitation, the {001} facet exhibits a higher photoelectron intensity compared to the {111} facet. This observation offers valuable insights into the anisotropic charge transfer.

### 2.2 Spatially Resolved Photoluminescence Mapping

**Figure 3:**
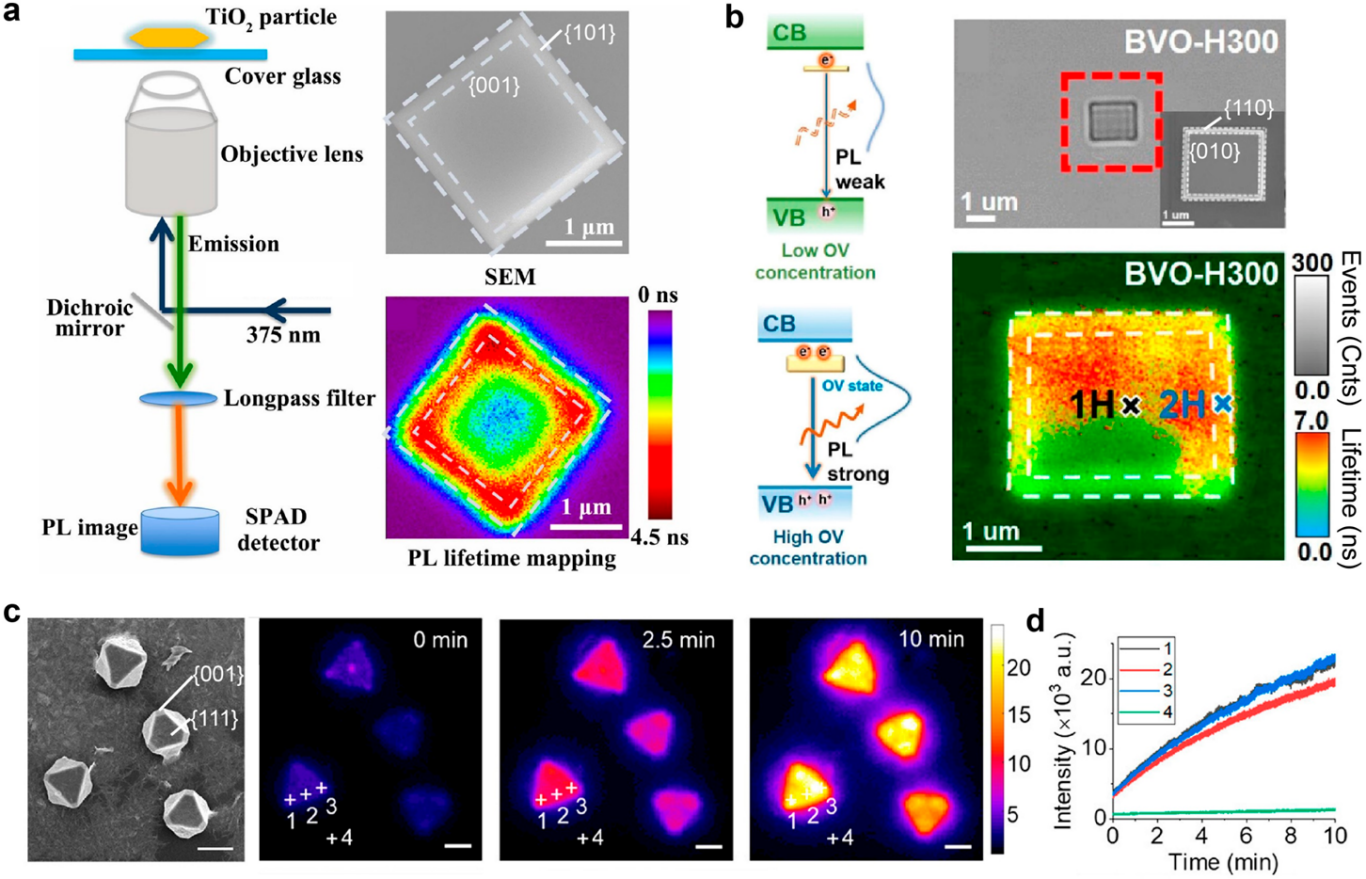
Spatially resolved photoluminescence mapping at the single particle level. (a) Single-particle photoluminescence measurements to investigate the dynamics of photogenerated electron/hole pairs on a single TiO_2_ particle. Reprinted with permission from ref (47). Copyright 2019, National Academy of Sciences. (b) In situ tracking the spatial distribution of oxygen vacancies across different facets of BiVO_4_. Reprinted with permission from ref (48). Copyright 2023, American Chemical Society. (c, d) The photocorrosion activity on a single Cu_2_O can be elucidated by monitoring the dynamic evolution of doubly ionized oxygen vacancies, singly ionized oxygen vacancies, and copper vacancies. The interfacial composition changes of Cu_2_O samples were investigated under hole scavenger (sodium ascorbate) and electron scavenger (potassium bromate). Scale bars: 2 μm. Reprinted with permission from ref (49). Copyright 2022, American Chemical Society.

Single-particle photoluminescence microscopy is a technique employed to measure the emission of photoluminescence from individual particles.^43−45^ By illuminating these particles with light of a specific wavelength, they can absorb energy and re-emit it in the form of light, a phenomenon referred to as photoluminescence.^46^ This microscopy method is valuable, as it uncovers heterogeneities of carrier dynamics and unique photochemical properties that may be concealed in ensemble measurements. By analyzing spatially resolved information such as photoluminescence spectra, intensity, and lifetime, important properties about individual particles (e.g., distribution of electronic defects and dynamics of charge carriers) can be deduced. For example, a recent study employed single-particle photoluminescence measurements to investigate the dynamics of photogenerated electron/hole pairs on a single TiO_2_ particle (Figure 3a).^47^ The authors observed shorter photoluminescence lifetimes at the center of the particle and longer lifetimes at the around-surface heterojunction of basal {001} and lateral {101} facets. Photoluminescence intensity also increased from the middle area to the edges and corners and was nearly the same on specific facets with similar spatial characteristics.

The presence of oxygen vacancies (OVs) on specific sites can enhance the interaction between reactants and oxide surfaces, facilitating interface charge transfer. However, accurately monitoring the spatial distribution of OVs remains a significant challenge. In a recent study, the single-particle spectroscopy technique was employed to in situ track the photoluminescence lifetimes and spectra of individual particles across different facets of BiVO_4_.^48^ The authors discovered a positive correlation between defects and bound exciton luminescence across different facets and observed that OVs were more likely to be generated at the {010} facets (Figure 3b, bottom right). As depicted in Figure 3b, after hydrogen treatment, photoluminescence lifetime mapping revealed that the basal {010} facet had a greater number of regions with longer lifetimes compared to the lateral {110} facet. Moreover, the rate of increase in photoluminescence lifetime on the {010} facet was significantly higher than that on the {110} facet. These differences between the {010} and {110} facets can be attributed to a greater generation of OVs on the {010} facet after hydrogen treatment, as OVs act as electron capture sites that impede charge recombination. The photoluminescence emission peak is approximately at 1.82 eV, which is lower than the ∼2.25 eV band gap, indicating that the photoluminescence originates from intraband states (Figure 3b, bottom left). Hence, a higher density of OVs leads to stronger emission. The photoluminescence intensity of the {010} facet in hydrogen-treated BiVO_4_ was found to be 140% higher than that of untreated BiVO_4_, whereas the {110} facet exhibited only a 47% increase over untreated BiVO4 (Figure 3b, bottom right). This evidence further supports the idea that OVs are more likely to be generated on the {010} facets. Consequently, such anisotropic defect engineering substantially prolongs carrier lifetimes.

Another study also utilized single-particle photoluminescence microscopy to investigate defects, revealing heterogeneous photocorrosion activity on Cu_2_O.^49^ The emission of photoluminescence photons at 615, 710, 820, and 900 nm arises from distinct recombination processes involving photoexcited electrons, valence band holes, and vacancies in Cu_2_O. Specifically, the 615 nm emission results from electron–hole recombination in the bands, the 710 and 820 nm emissions arise from doubly ionized (VO^2+^) and singly ionized (VO^+^) oxygen vacancies, and the 900 nm emission stems from copper vacancies (V_Cu_). Upon exposure to 647 nm light, the corner locations of the Cu_2_O microcrystals exhibit significantly enhanced photoluminescence compared to the edge and face sites, resulting in a triangular pattern with intensified brightness at the corner points (Figure 3c,d). This increased photooxidation at the corners, relative to the edges and faces, may be attributed to the greater instability of the (100) facet compared to other regions. In a system with an electron–hole scavenger, photooxidation and photoreduction occur concurrently but unevenly on the Cu_2_O surface, and the rate of copper vacancy formation is the highest among these processes.

### 2.3 Photoelectrochemical Kinetics at the Single or Subparticle Level

Single-molecule fluorescence microscopy has emerged as a powerful tool for investigating photocatalytic processes under operando conditions, providing nanoscale spatial resolution and millisecond temporal resolution.^50−52^ By leveraging the detection of fluorescence emitted by individual fluorophores attached to the photocatalyst, this technique enables the visualization and analysis of catalytic events at the single-molecule level.^53,54^ Nonfluorescent or weakly fluorescent substrate molecules are introduced into the reaction system during photocatalysis. As the photocatalytic reaction occurs, the substrate molecules are transformed into highly fluorescent product molecules.^55^ Through the identification of individual fluorescent molecule and reconstruction of numerous catalytic events, quantitative information (including reaction sites, kinetics, and behaviors of charge carriers on the surface of individual photocatalysts) can be obtained.^56−58^

**Figure 4:**
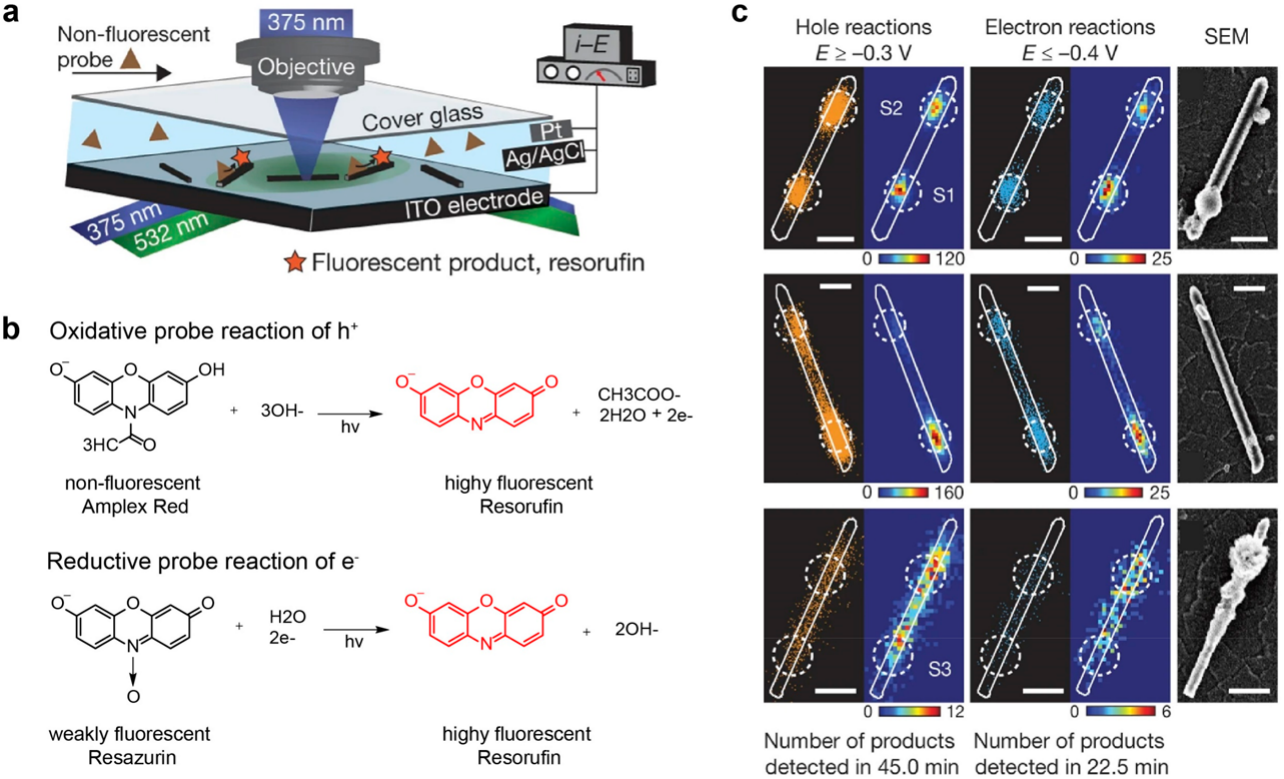
Spatially resolved mapping of surface electron and hole activity at a single nanorod. (a) Dual-laser wide-field epifluorescence illumination and a microfluidic liquid cell were used for single-molecule localization imaging. (b) Reaction mechanisms of oxidative and reductive fluorogenic probes. (c) Two-dimensional histogram of all fluorescent product molecules generated from hole-induced amplex red oxidation and electron-induced resazurin reduction on TiO_2_ nanorods. Bin size: 40 by 40 nm. The bin size represents the area of each bin in the two-dimensional histogram of all individual resorufin product molecule. Scale bars: 400 nm. Reprinted with permission from ref (59). Copyright 2016, Springer Nature.

The spatial distributions and dynamics of photogenerated electrons and holes on a single photocatalyst can be accurately resolved through a charge carrier-selective imaging protocol.^59^ This protocol involves the use of a customized electrochemical flow cell and a dual-laser wide-field epifluorescence illumination setup. The 405 nm laser is utilized to excite the semiconductor, while the 532 nm laser is employed to excite the fluorescent product. This setup closely replicates the reaction conditions in practical photocatalysis reactors (Figure 4a). By selectively switching the redox properties of fluorogenic molecules, both electron-induced and hole-induced reactions can be achieved. The oxidative probe reaction for holes involves the transformation of the nonfluorescent amplex red (AR) into the highly fluorescent product resorufin (P), while the reductive probe reaction for electrons entails the transformation of the weakly fluorescent resazurin (Rz) into the same fluorescent product resorufin (Figure 4b). The center position of individual fluorescent P products can be accurately determined using 2D Gaussian point spread function (PSF) fitting. This technique achieves a high localization precision of single molecule events, reaching up to 30 nm.

TiO_2_ nanorods with well-defined {100} side facets, measuring 127 ± 27 nm in width and 1735 ± 437 nm in length, were utilized as a model system to investigate the reactivity of charge carriers. Oxygen evolution catalysts (OEC) were deposited onto the nanorods using a focused 375 nm laser with nanoscale precision under imaging mode. Super-resolution images obtained through correlated scanning electron microscopy (SEM) were then compared with corresponding SEM images of the same photocatalyst to reveal the structure–reactivity relationship at the spatial level. Figure 4c demonstrates that a majority of the tested nanorods (∼68%) exhibit nonuniform reactivity of holes and electrons, characterized by a few nanoscale “hotspots”. However, the remaining nanorods display delocalized charge carrier reactivity along the {100} side facets. This intraparticle heterogeneity suggests that the presence of surface structural defects (confirmed as Fe impurities through elemental analysis) may contribute to the high reactivity sites, rather than the influence of specific facets. Interestingly, super-resolution imaging clearly indicates that holes and electrons tend to react at the same site on each individual nanorod. The close proximity of hole- and electron-induced hotspots on the same nanorod, with an average separation distance of only 40 nm, emphasizes the importance of carefully adjusting the distribution of electrons and holes to mitigate charge carrier recombination in this type of photocatalyst.

Surface defects is an important factor that can contribute to the local charge separation and reactivity. A separate study on single molecule imaging of Sb-doped TiO_2_ nanorods suggests that the two ends of the nanorod exhibit higher reactivity compared to the middle section over a long testing period (up to 13 h).^60^ The time-lapsed 2D histogram analysis of fluorescent product molecules during the reaction of Sb-doped TiO_2_ nanorods indicates the existence of a fluctuation in spatial reactivity: middle section has higher transient activity initially but deactivates rapidly. The dynamic spatiotemporal fluctuations of reactivity patterns observed in the Sb-doped TiO_2_ nanorods can indeed be attributed to the reaction-driven surface reconstruction at the middle section, particularly involving unstable defect sites. The reactivity of hole and electron can also be quantitively studied by detecting hydroxyl (OH•) and superoxide anion radicals (O_2_^–•^) using AR probes. According to the Langmuir–Hinshelwood equation, the equilibrium adsorption constant for the reaction with OH^•^ is reported to be 3 to 4 times larger than the reaction with O_2_^–•^. This finding suggests that the positively or negatively charged microenvironment around the reaction sites can greatly affect the binding/dissociation process of substrate/product molecules and subsequent electron transfer processes.

The two fluorogenic probe colocalization method provides a promising approach to elucidate the relationship between surface defects and reactivity on a single photocatalyst.^61,62^ By employing hydroxyl radical-selective probe (3′-(*p*-aminophenyl)fluorescein, APF) and furfuryl alcohol probe (FA) to detect surface defects such as oxygen vacancies, it becomes feasible to establish a clear structure–activity relationship at the single-molecule level. Remarkably, a strong spatial correlation is observed between hydroxyl radical and oxygen vacancy-induced reactions on each nanorod. The treatment of W_18_O_49_ nanowires with ascorbic acid, resulting in the creation of uniform oxygen vacancies, can lead to a significant improvement in reactivity. Specifically, the rate constant for the generation of hydroxyl radicals (•OH) increases from 248 μm^–1^ min^–1^ to 442 μm^–1^ min^–1^.

**Figure 5:**
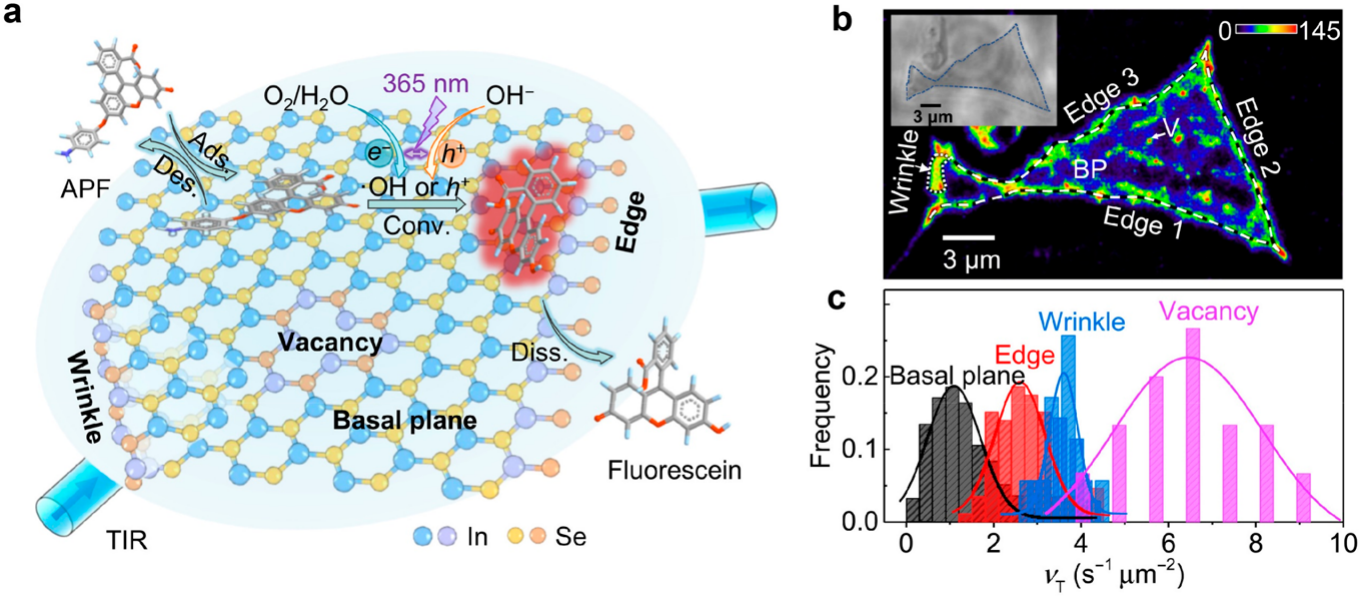
Quantification of the heterogeneous photocatalytic activities on two-dimensional layered materials. (a) The hole-induced oxidization reaction was probed by APF probe under a total internal reflection configuration. (b) Mapping of photocatalytic events on InSe flakes. Bin size: 24 by 24 nm. The color bar denotes the number of detected fluorescent product molecules. (c) Histogram distribution of photocatalytic turnover rates at four kinds of typical subregions. Reprinted with permission from ref (63). Copyright 2021, American Association for the Advancement of Science.

Single-molecule fluorescence imaging techniques provide exceptional capabilities for detecting individual turnover events with high spatial resolution at the nanometer scale. This enables the quantification of catalytic turnover rates at specific subregions on a photocatalyst. By utilizing APF probes, it is possible to observe the oxidation of these probes into highly fluorescent molecules, such as fluorescein, upon excitation by electrons or holes using a 488 nm laser in a total internal reflection (TIR) configuration (Figure 5a).^63^ Specific catalytic turnover rate (*V*_T_) can be calculated by counting the single turnover events at each subregion of the InSe flakes. Higher photocatalytic activities at edge (2.6 ± 0.6 s^–1^ μm^–2^), wrinkle (3.6 ± 0.3 s^–1^ μm^–2^), and vacancy (6.5 ± 2 s^–1^ μm^–2^) regions can be observed compared to the basal plane (1.1 ± 0.6 s^–1^ μm^–2^, Figure 5b) of InSe flakes. The quantitative study confirms that photocatalytic active sites with defects exhibit lower adsorption strength with reactants and slower dissociation/diffusion rates of products compared to the basal plane without defects. A similar trend of photocatalytic activities can be observed in 2D g-C_3_N_4_ nanosheets, where higher activities are observed at the wrinkles (22.6 ± 2.8 s^–1^ μm^–2^) and edge (10.9 ± 1.4 s^–1^ μm^–2^) regions compared to the basal plane (2.8 ± 0.5 s^–1^ μm^–2^).^64^ The conversion rate constant and adsorption equilibrium constant can be solved according to the Langmuir–Hinshelwood kinetic model. The wrinkle sites in 2D g-C_3_N_4_ nanosheets exhibit the highest chemical conversion rate (139 ± 2.3 s^–1^ μm^–2^), which is 24% and 69% higher compared to the edges and basal plane, respectively. The basal plane sites in 2D g-C_3_N_4_ nanosheets exhibit the highest adsorption equilibrium constant (23.4 ± 1.0 μM^–1^), which is around 19% higher compared to the edges and wrinkle, respectively.

**Figure 6:**
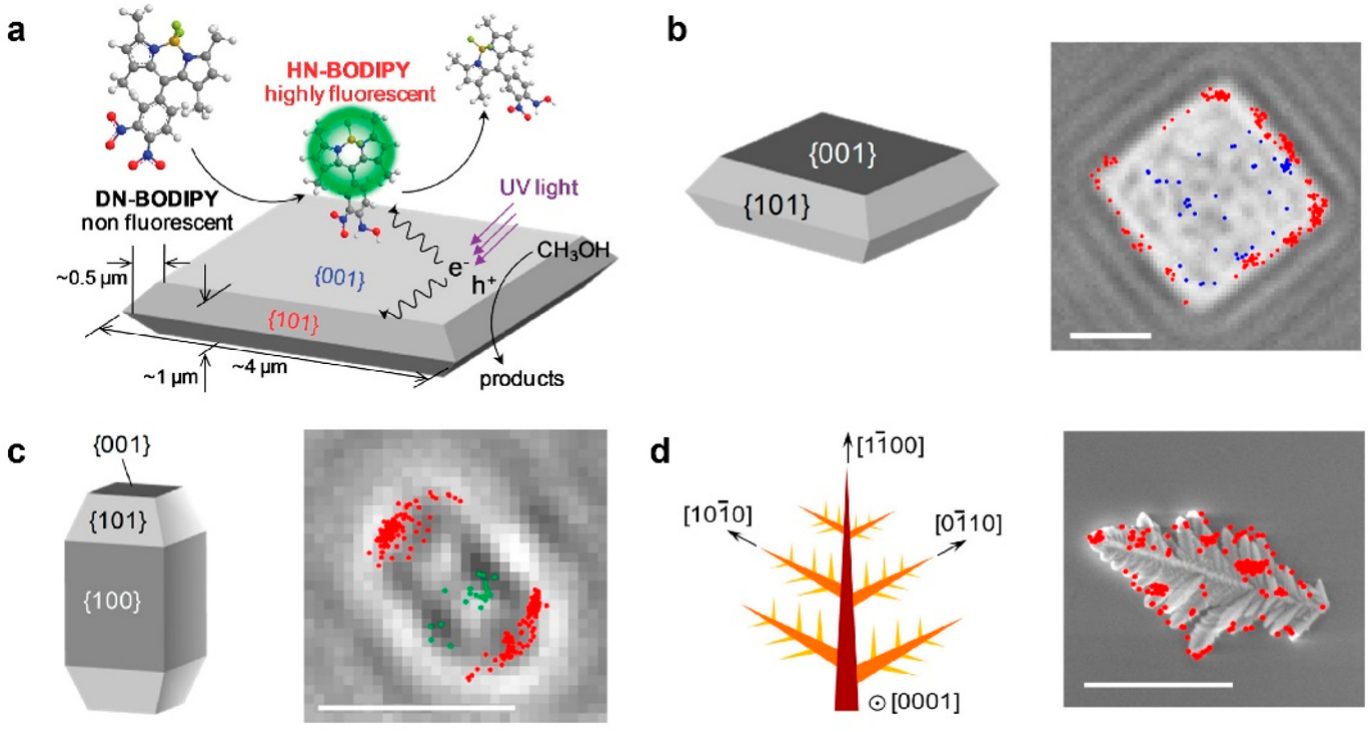
Facet-dependent photocatalytic activities on an anisotropically shaped photocatalyst particle. (a) Single-molecule fluorescence imaging for resolving the reactivity heterogeneity at different facets. Reprinted with permission from ref (65). Copyright 2011, American Chemical Society. (b) Locations of the reactive sites on TiO_2_ plate: blue dots for (001) surfaces and red dots for (101) surfaces. Scale bars: 2 μm. (c) Locations of the reactive sites on TiO_2_ nanorod: green dots for (001) surfaces and red dots for (101) surfaces. Scale bars: 2 μm. (d) Locations of the reactive sites on an α-Fe_2_O_3_ micropin. Scale bars: 2 μm. Reprinted with permission from ref (66). Copyright 2013, American Chemical Society.

Single-molecule fluorescence imaging has proven to be an effective tool in resolving the reactivity heterogeneity at different facets, even at the subfacet level. In a study conducted by Tachikawa et al., they employed a redox-responsive fluorogenic dye called 3,4-dinitrophenyl-BODIPY (DN-BODIPY) to investigate the inherent photocatalytic activity of anatase-exposed surfaces of TiO_2._^65,66^ By utilizing this technique, they were able to elucidate the variations in photocatalytic activity among different surface facets of TiO_2_. This study uncovered that the {101} facets of TiO_2_ crystals contain the active catalytic sites that are essential for the effective reduction of probe molecules, even though the {001} facets have higher surface energy. (Figure 6a). This unexpected finding can be attributed to the directional flow of photogenerated charge carriers toward specific surfaces. To identify effective adsorption sites on metal oxide surfaces, researchers employed single-molecule fluorescence microscopy using a catechol-modified boron-dipyrromethene dye called CA-BODIPY. Upon adsorption of CA-BODIPY molecules onto TiO_2_ nanoparticles, distinct fluorescence signals were observed, indicating the formation of chelating complexes between the catechol moiety of CA-BODIPY and surface Ti sites on the nanoparticles. In a single TiO_2_ microcrystal primarily composed of {001} facets, the fluorescence spots were notably concentrated on the lateral {101} facets, emphasizing the influence of crystal face orientation on adsorptive capacity (Figure 6b). The investigation was extended to include the {100} facets, and the collected data from TiO_2_ crystals predominantly featuring {100} facets established an adsorption hierarchy as follows: {101} > {001} ≈ {100} (Figure 6c). The study also explored the adsorption sites on α-Fe_2_O_3_ dendritic micropines. These micropines exhibit a dendritic structure aligned in the [0001] direction, branching off into the [101̅0], [11̅00], and [01̅10] directions. It was observed that CA-BODIPY molecules preferentially adsorb at the apex of α-Fe_2_O_3_ micropine branches, likely due to the expected high density of exposed iron cations in those regions (Figure 6d).

**Figure 7:**
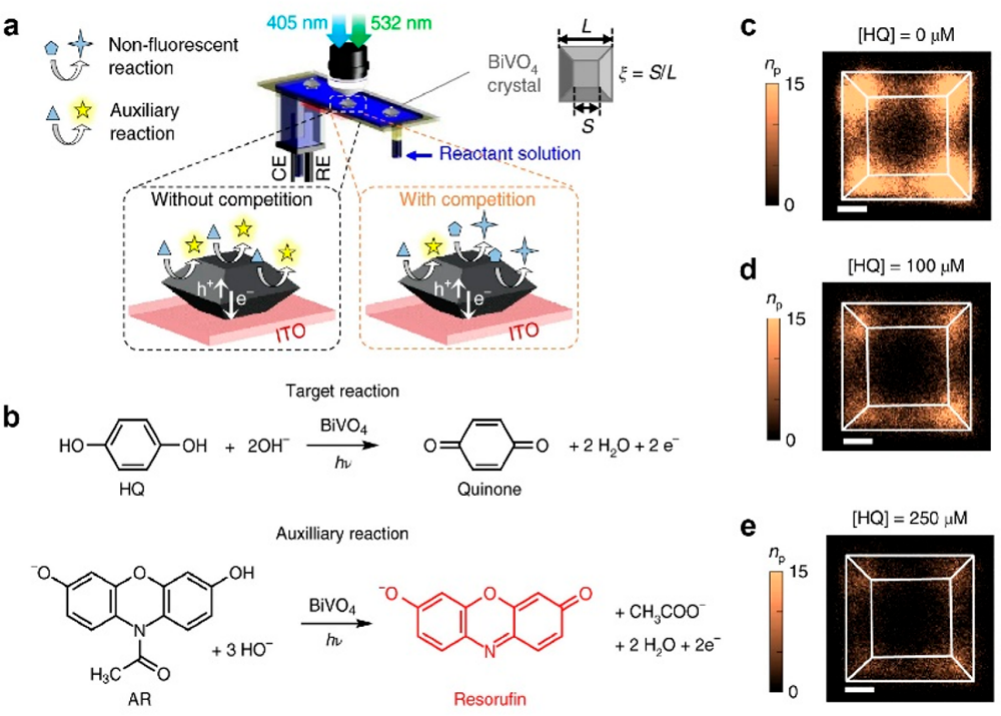
Spatially resolved photoelectrocatalytic activities of single particles toward nonfluorescent reactions via competitive chemistry-based super-resolution microscopy. (a) COMPEITS imaging is based on competition between two reactions. (b) Nonfluorescent target reactions (hydroquinone oxidization) and auxiliary fluorogenic reactions (amplex red oxidization) are introduced in COMPEITS imaging. (c–e) Two-dimensional histogram of all fluorescent product molecules generated from auxiliary fluorogenic reactions with 0 μM (c), 100 μM (d), 250 μM (e) hydroquinone. Bin size: 40 by 40 nm. Scale bars: 500 nm. Reprinted with permission from ref (67). Copyright 2019, Springer Nature.

The previously mentioned super-resolution imaging experiments rely on fluorogenic reactions using fluorescent probes, which may not be applicable to target reactions that do not involve fluorescent intermediates or products (e.g., water splitting, N_2_ reduction, CO_2_ reduction). To overcome this limitation, a novel technique called Competition-Enabled Imaging Technique with Super-Resolution (COMPEITS) has been developed.^67,68^ In a COMPEITS imaging experiment, both the nonfluorescent target reactant and an auxiliary fluorogenic reactant are introduced into the reaction system and allowed to compete at the same active sites on individual nanoparticles (Figure 7a). The degree of inhibition of the fluorogenic reactions directly reflects the reaction sites and kinetics of the target reaction due to the competition between the two reactions. To investigate the hydroquinone (HQ) oxidation, which is important for the degradation of phenolic micropollutants in aquatic ecosystems, AR oxidation was chosen as the auxiliary fluorogenic reaction (Figure 7b). In the absence of hydroquinone, the number of resorufin product molecules (*n*_p_) and the AR oxidation rate can be quantified with a spatial resolution of 40 nm using conventional data analysis methods in super-resolution imaging. As the concentration of HQ increases, a decrease in the number of detected AR oxidation products is observed. The inverse difference (Δ*n*_p_^–1^) shows a linear relationship with the adsorption equilibrium constant of HQ, allowing the COMPEITS images to be quantitatively related to the binding affinity of HQ on BiVO_4_ semiconductors.

### 2.4 Photocurrent Mapping

**Figure 8:**
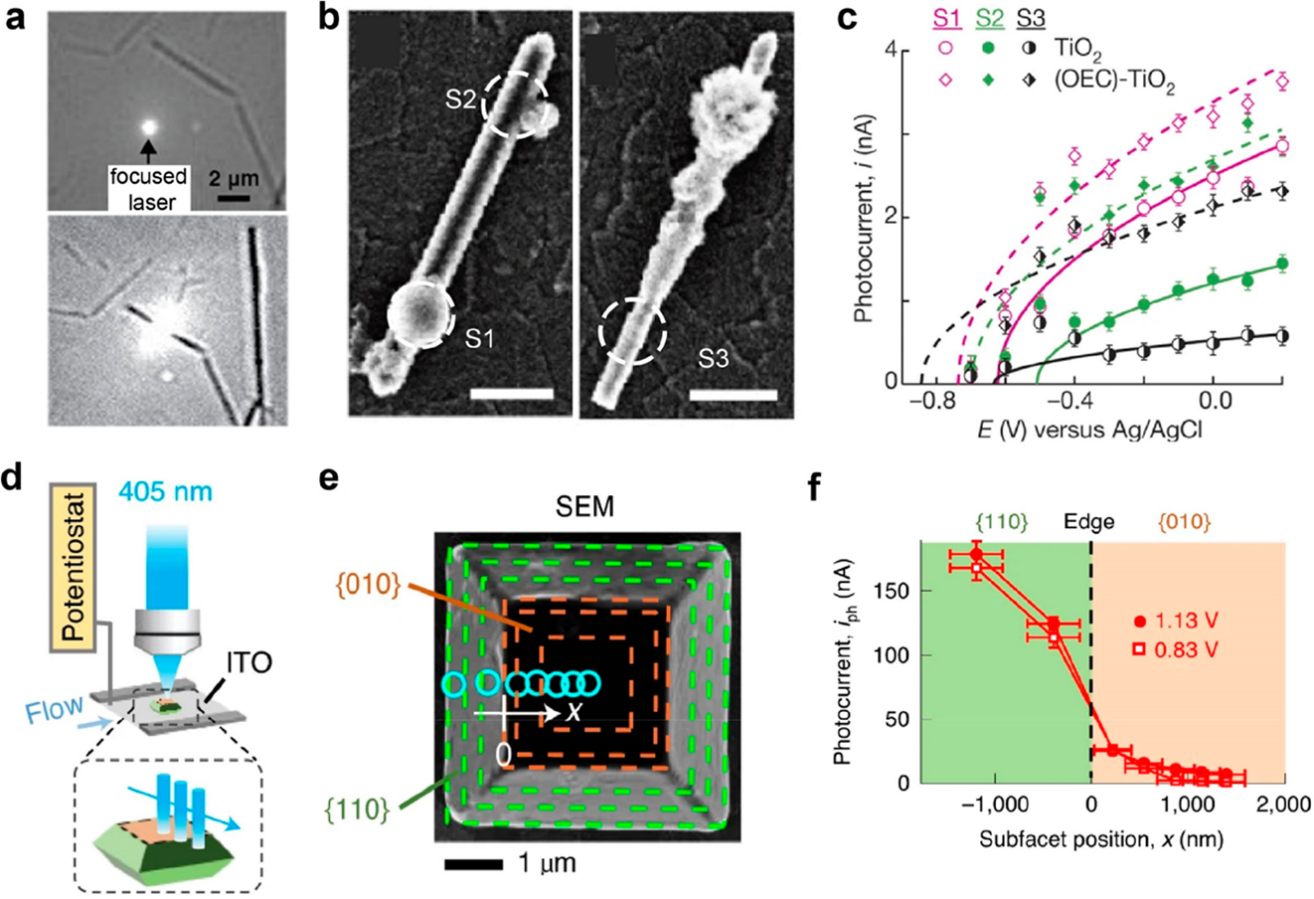
Focused laser illumination to reveal the heterogeneous photocurrent at subparticle level. (a) Diffraction-limited laser spot on ITO electrode or TiO_2_ nanorod for spatially resolved photocurrent measurement. (b) SEM images of the three TiO_2_ nanorods decorated with oxygen evolution catalyst (OEC). Scale bars: 400 nm. (c) Local i–E responses at different locations (S1, S2, and S3 in Figure 8b) with or without OEC deposition. Reprinted with permission from ref (59). Copyright 2016, Springer Nature. (d) Focused laser excitation for photocurrent mapping along {010} and {110} facets of BiVO_4_ particles. (e) SEM image of anisotropically shaped BiVO_4_ particles. Circles represent the position and size of the focused laser spot. (f) Subfacet position-dependent photoelectrochemical current curve exhibits an *s*-like transition. Reprinted with permission from ref (69). Copyright 2022, Springer Nature.

Photocurrent is a crucial parameter for directly evaluating the overall reactivity and efficiency of solar-driven reduction/oxidation processes in ensemble-level tests. The recently developed photocurrent microscopy approach, as demonstrated in Figure 8a, offers a means to acquire spatially resolved photocurrent information by scanning a diffraction-limited laser spot across the sample surface.^59^ A focused 375 nm laser (∼390 nm in diameter) is utilized to locally excite the charge carriers on the TiO_2_ nanorod. Concurrently, the current changes of the indium tin oxide (ITO) working electrode are measured. These current changes are indicative of the local performance parameters, such as electron–hole separation efficiency and water oxidation kinetics. The local photocurrents on a single nanorod are enhanced sharply after OEC deposition, and the photocurrents at different sites (such as S1, S2, and S3 in Figure 8b,c) follow a scaling relationship with the square root of the potential (*E*). The spatially resolved absorbed-photon-to-current efficiency (η) and hole and electron surface activities (*k*_h_ and *k*_e_) can be extracted according to the Gärtner–Butler and Reichman models. The strong correlation among η, *k*_h_, and *k*_e_ suggests that both the oxidation and reduction reactions, involved in water oxidation and reduction, occur with greater efficiency at the same site under bias.

Photocurrent microscopy approach can also be used to uncover the heterogeneous reactivity across the interfacet junction on an anisotropically shaped photocatalyst particle.^69^ BiVO_4_ with a truncated bipyramid morphology, exposing {010} and {110} facets, was chosen as a model system (Figure 8d–f). The subfacet position-dependent photocurrent curve shows a s-shaped increase from the {010} facet, interfacet edge to the {110} facet, instead of a sudden change at the near-edge region. The existence of this micrometer-scale transition zone and the gradual increase in photocurrent across the interfacet junction indicate the significance of interfacet junction effects in the design of photocatalysts. It suggests that the reactivity and efficiency of the photocatalyst are influenced not only by the individual facets but also by the interactions and effects at the junctions between different facets.

**Figure 9:**
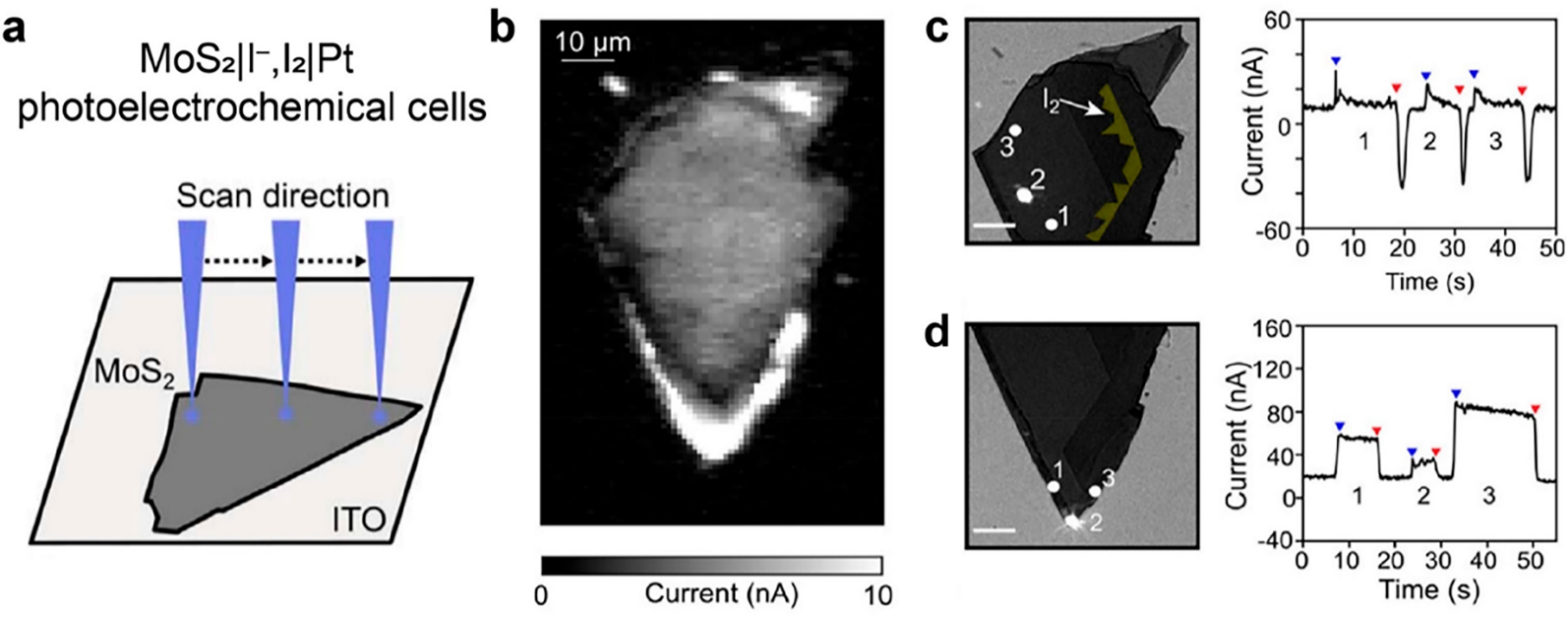
Photoelectrochemical reactivity mapping of MoS_2_ nanoflakes through local iodide oxidation reaction. (a) Scanning laser spot technique for an illumination position-dependent photocurrent mapping. (b) Photocurrent mapping of the MoS_2_ nanoflake. Reprinted with permission from ref (70). Copyright 2018, American Chemical Society. (c, d) Photogenerated carriers induced I_2_ deposition and corresponding current vs time curves upon illuminating local regions. Reprinted with permission from ref (71). Copyright 2018, American Chemical Society.

While the photocurrent microscopy approach is useful for assessing charge carrier activity through local-region illumination, it may not directly reveal the specific origin and location of charge carrier-induced reactions within the semiconductor particle. However, by incorporating an iodide oxidation reaction into the photocurrent microscopy approach, it becomes possible to resolve the location of photogenerated products (I_2_), by determining the local optical density change under bright field imaging mode (Figure 9a).^70,71^ Photocurrent mapping of MoS_2_ nanoflakes can be obtained by a conventional photocurrent microscopy approach using a focused 532 nm laser spot (690 nm in diameter). The obtained photocurrent mapping reveals nonuniformity in the photocurrents at the perimeter edges of the MoS_2_ nanoflake. Some regions exhibit a photocurrent up to 10 nA, while other regions show negligible or significantly lower photocurrent. During local photoelectrochemical measurements on the basal plane (spots 1–3 in Figure 8c), the accumulation of I_2_ products at the step edge sites becomes apparent in the bright field transmission images (indicated by false yellow color pixels). On the basis of theoretical calculations regarding the critical formation rate on the MoS_2_ nanoflake, it is inferred that nearly all photogenerated holes are transported to the step edge sites and react with iodide to form I_2_. Interestingly, no detectable I_2_ formation is observed when laser spots are focused on the perimeter edges, despite the higher photocurrents compared to the basal planes. This discrepancy suggests the possibility of different reaction pathways or kinetics occurring at the perimeter edges in comparison to the basal planes. One potential explanation is that the transport distances of charge carriers are greater than several tens of micrometers, and the local rate of iodine formation may not reach the critical rate required for iodine deposition.

## 3 Functional Imaging Guided Photocatalyst Designs

The functional imaging techniques discussed in section 2 have proven highly effective in exploring and comprehending various aspects of semiconductor materials at the single-particle level. These techniques enable the investigation of heterogeneous catalytic activity, interface defect states, surface potentials, local photocurrent, and more. Consequently, they contribute significantly to constructing a comprehensive local structural profile of semiconductor materials, providing valuable insights into their properties and behavior. Moreover, the application of functional imaging allows for the simultaneous study of a large number of catalyst particles at the single-particle level. This capability facilitates the quantitative screening of their local activity, providing valuable data for understanding the intricate relationships between catalyst structure and activity. In this section, we will highlight several studies that have utilized imaging-guided approaches to offer design principles for high-performance photocatalysts. These studies shed light on strategies for optimizing catalyst structures and properties, leveraging the power of functional imaging techniques to advance the field of semiconductor photocatalysis.

### 3.1 Particle Size/Shape Engineering

**Figure 10:**
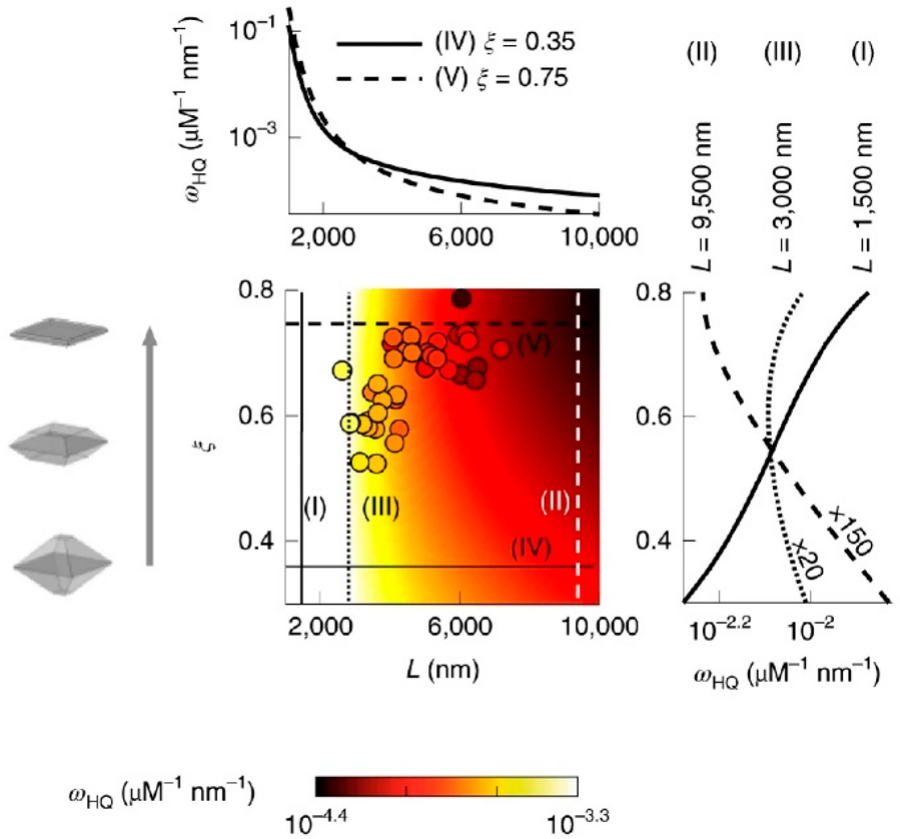
The particle size/shape scaling law governs reactant adsorption efficacy on BiVO_4_ photocatalysts. Reprinted with permission from ref (67). Copyright 2019, Springer Nature.

The chemisorption of the nonfluorescent reactant molecule HQ at superoptical resolution can be imaged and quantified, thus enabling deconvolution of both the size (parameter *L*) and shape (parameter ξ) on the properties of specific facets of BiVO_4_ single catalyst particles (Figure 10).^67^ The anisotropic spatial distribution of adsorption efficacy relies on both size and shape. For any BiVO_4_ particle, regardless of the size and shape, the HQ adsorption efficacy of the basal {010} is almost two times larger than the of lateral {110} facets. At any fixed shape (constant ξ), HQ adsorption efficacy on both facets decrease asymptotically with increasing *L*, due to the size-dependent surface energy. The significant edge effects are also found besides the facet-dependent spatial distribution. In detail, edge regions between the basal and lateral facets (type-I edges) and edge regions between the lateral facets (type-II edges) show distinct features, compared with the basal facet or lateral facet, and type-I edge on average having a larger HQ adsorption efficacy. As a result, the HQ adsorption efficacy on both facets is also decreased asymptotically as the particle shape transitions from bipyramid-like to plate-like (i.e., with increasing ξ). Decoupling the size and shape effects of adsorption efficacy enable the rational design of photocatalysts. To optimize the most efficient photocatalysts, the unit-mass-level whole-particle adsorption efficacy (ω_HQ_) of HQ molecular is defined as a parameter to evaluate the efficacy. Shape-related ω_HQ_ exhibits three distinct types. In detail, for intermediate sizes (∼2.3 μm < *L* < ∼9 μm), either plate-like or bipyramid-like exhibit the maximal ω_HQ_, instead of a truncated bipyramid. For smaller ones (*L* < ∼2.3 μm), plate-like possesses larger ω_HQ_. For larger ones (*L* > ∼9 μm), a bipyramid-like shape (smaller ξ) is better.

### 3.2 Chemical Manipulation of Photocatalysts

**Figure 11:**
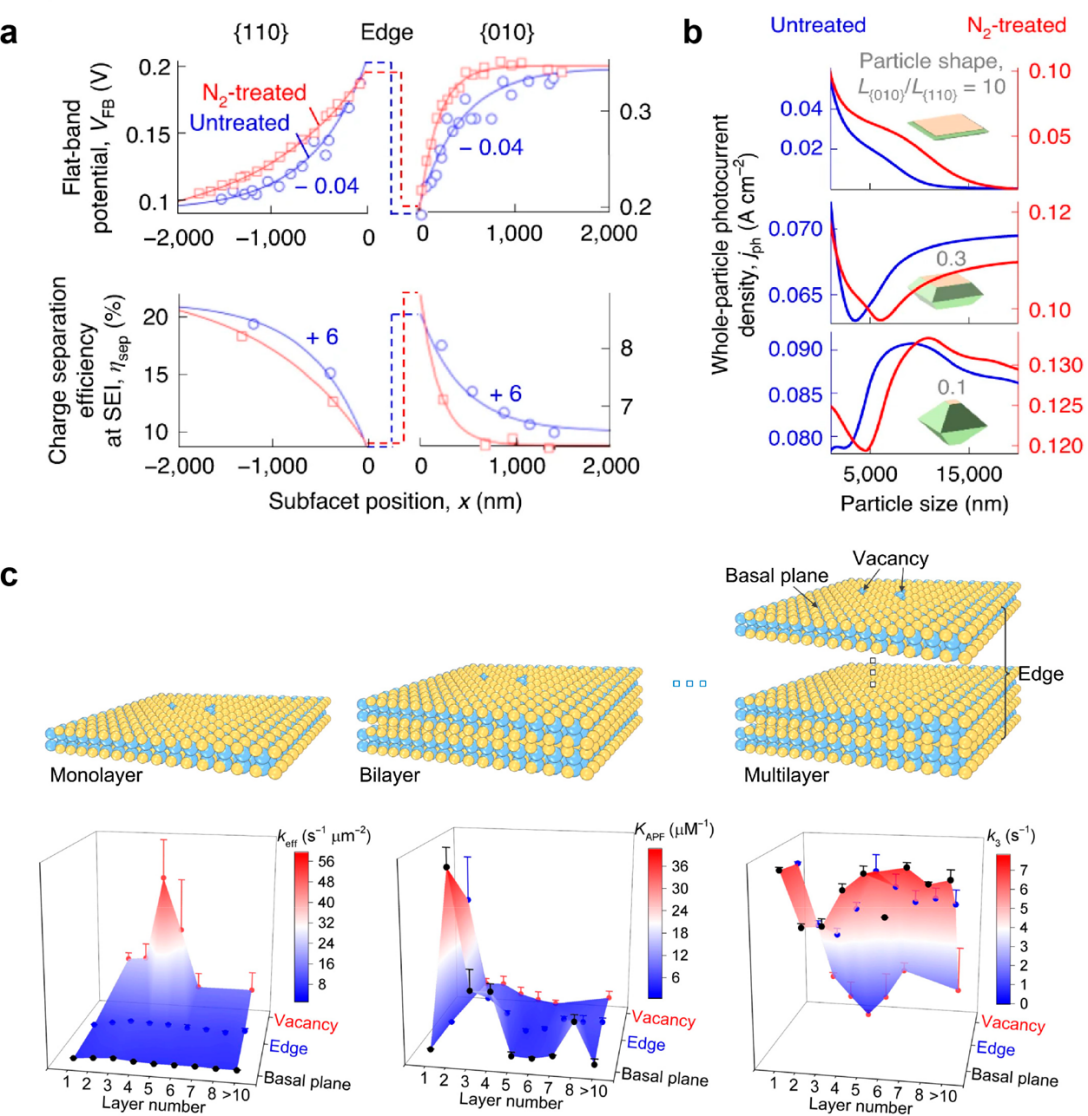
Chemical modifications to modulate photoelectrode performance. (a) *V*_FB_ (top) and η_sep_ (bottom) differences of BiVO_4_ photocatalysts with or without N_2_ treatments. (b) Interfacet junction effects governed whole-particle photoelectrochemical performance. Reprinted with permission from ref (69). Copyright 2022, Springer Nature. (c) Layer-dependent photoelectrochemical reactivity at basal planes, edges, or vacancy sites of 2D layered InSe. Reprinted with permission from ref (63). Copyright 2021, American Association for the Advancement of Science.

Modifying the surface dopant or defect densities is a widely used approach for adjusting the performance of photoelectrodes.^72^ By combining single molecule imaging with local photocurrent measurement techniques, we can effectively discern variations in local functional parameters, such as flat-band potential (*V*_FB_) and electron–hole separation efficiency (η_sep_), following chemical doping.^69^ BiVO_4_ particles were thermally annealed in nitrogen to introduce nitrogen elements and oxygen vacancies at the surface. Untreated and N_2_-modified particles with similar size and shape were used for single particle measurements. As shown in Figure 11a, particles treated with N_2_ display more significant variations on the donor-rich {010} facets as they move away from the interfacet edge, indicating narrower near-edge surface transition zones. Conversely, on the acceptor-richer {110} facets, a contrasting trend is observed: shallower variations imply broader near-edge surface transition zones. The modified widths of the near-edge surface transition zones in the N_2_-treated particles disrupt the facet-size-dependent behavior of whole-particle photoelectrochemical current densities (as discussed in section 3.1). The photoelectrode performance of specifically shaped particles with different length ratios between {010} and {110} facets (*L*{010}/*L*{110) and facet composition doping can be predicted based on the combined effects of doping and particle size dependence (Figure 11b). For {010}-dominated plate-like particles, the whole-particle photoelectrochemical current densities show a consistent decrease as the overall particle size increases. The impact of doping on this monotonic decrease with increasing overall particle size is not significant. In the case of truncated bipyramid-like particles with a higher {110} fraction (*L*{010}/*L*{110} = 0.3), the whole-particle photocurrent density exhibits a biphasic behavior with the increasement of particle size. As the particle size further increases, the facet composition effect becomes dominant. In the case of {110}-dominated bipyramid-like particles (*L*{010}/*L*{110} = 0.1), a triphasic behavior observed in the photocurrent density. The N_2_ treatment induces changes in facet composition and the width of near-edge surface transition zones, leading to a shift in the optimal particle size for maximizing the photocurrent density. Specifically, for the untreated samples, the optimal particle size is 8.3 μm, whereas for the N_2_-treated samples, it shifts to 10.9 μm. These changes in facet composition and near-edge surface transition zones play a crucial role in determining the optimal particle size for achieving maximum photocurrent density.

Besides chemical doping in particulate semiconductors, defect states and thickness are two key factors that can influence the catalytic properties of 2D materials. The presence of defects can affect surface reactivity and electronic structure, while the thickness can impact the accessibility of active sites and the diffusion of reactants and products.^63^ To quantify the layer-dependent photocatalytic kinetics of materials such as InSe, parameters such as *k*_eff_ (chemical conversion rate constant), *K*_APF_ (adsorption equilibrium constant), and *k*_3_ (dissociation rate constant) can be determined through single molecule imaging. After analyzing the data collected from over 40 InSe flakes, consisting of more than 1000 basal planes, over 300 edges, and over 50 vacancies, it was observed that vacancies in 3–4 layered InSe exhibited the highest activity, indicated by the largest *k*_eff_ value (Figure 11c). Conversely, the basal planes of InSe were consistently found to be inert. The limited reactivity observed in 2D InSe with a thickness exceeding 8 layers can be attributed to its direct band structure, which closely resembles that of bulk InSe. Adsorption strength of APF (the reaction probe) also show a layer-dependent trend: 2–3 layered InSe show higher *K*_APF_ and moderate *k*_3_. This case study provides valuable insights into the utilization of functional imaging at the single-molecule level to inform the design of defect structures and facilitate precise control over the thickness of 2D semiconductors.

### 3.3 Control over Illumination Modes

**Figure 12:**
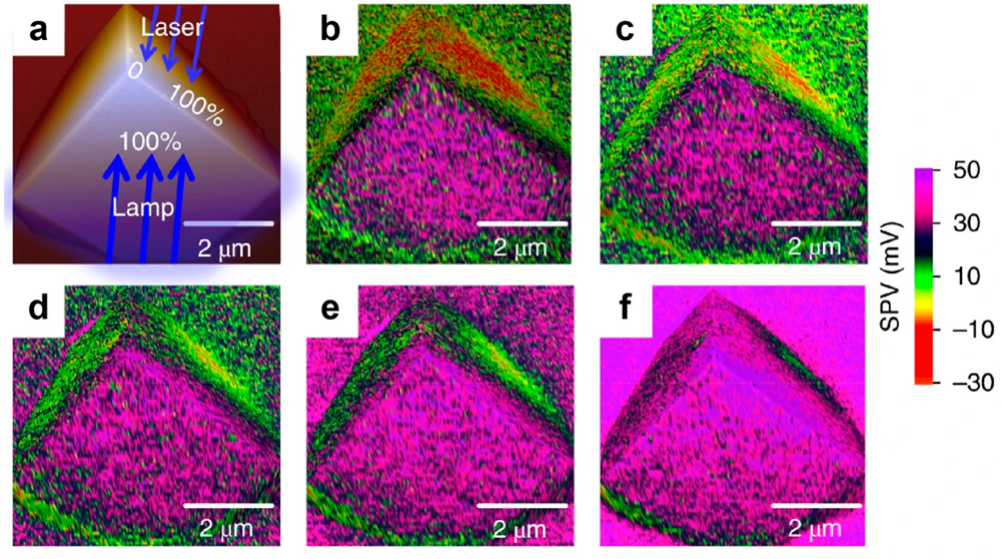
Investigation of single-particle charge separation under asymmetrical illumination. (a) Schematic representation of asymmetrical illumination on AFM topography image of a Cu_2_O particle. (b–f) The corresponding SPVM images of the same particle under different illumination types. The laser intensity is systematically adjusted to different percentages, specifically 0% (b), 10% (c), 20% (d), 40% (e), and 100% (f) of the lamp intensity. This variation in laser intensity allows for the examination of the illumination symmetry effects on charge separation within the particle. Reprinted with permission from ref (73). Copyright 2018, Springer Nature.

The illumination mode can also influence the charge separation in a semiconductor particle. SPVM, in combination with asymmetric illumination, reveals that the diffusion-dominant charge transfer process, driven by the difference in the mobilities of photogenerated electrons and holes, significantly contributes to charge separation. This finding expands our understanding of the factors influencing charge separation, going beyond the conventionally considered built-in electric fields (Figure 12a).^73^ To investigate this phenomenon, a single cubic Cu_2_O particle with two facets of the same Miller index is subjected to asymmetric illumination using two light sources: a laser and a xenon lamp. The choice of a cubic morphology aims to minimize intrinsic differences, such as built-in electric fields, between the illuminated and shadow facets. By increasing the laser intensity while keeping the xenon lamp intensity constant, significant variations in SPV are observed on the illuminated facets. Initially, without laser illumination, photogenerated holes accumulate on the front facet under direct illumination by the xenon lamp, while photoelectrons appear on the back shadow facet, as indicated by the positive and negative SPV values, respectively. As the laser power directed at the shadow facet increases, the accumulation of electrons gradually diminishes until all facets exhibit positive SPV values, indicating the presence of holes throughout the photocatalyst particle under symmetrical illumination. Quantitative analysis of the averaged SPV values in the center region of the illuminated and shadow facets reveals a gradual reduction in the divergence between the two facets as the degree of illumination asymmetry decreases. This experimental approach provides valuable insights into the interplay between asymmetric illumination and charge transfer dynamics, shedding light on the diffusion-driven charge separation process in photocatalysts.

### 3.4 Cocatalyst Engineering

**Figure 13:**
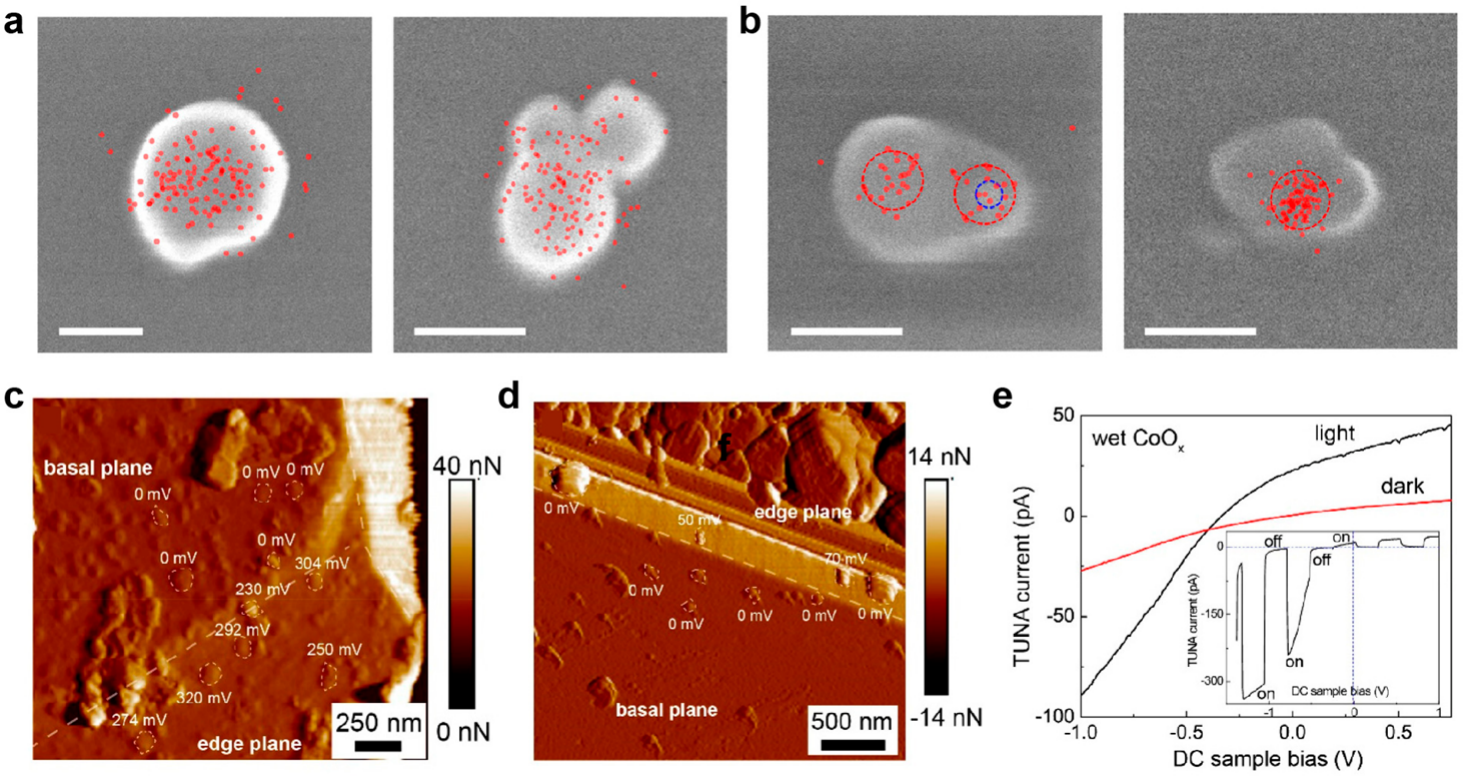
Spatial control over cocatalyst modifications on semiconductor particles. (a, b) Spatial distributions of probe-induced fluorescence spots on TiO_2_ (a) and 14 nm Au-decorated TiO_2_ particles (b). Scale bars: 100 nm. Reprinted with permission from ref (78). Copyright 2013, American Chemical Society. (c, d) Spatially resolved photovoltage maps obtained from local current-bias curves for decorating cocatalysts CoO_*x*_ (c) and Pt (d) nanoparticles on BiVO_4_, which are derived from local current–bias curves. (e) Tunneling AFM current versus DC sample vias curves on wet CoO_*x*_ nanoparticles under illumination or in the dark state, with the inset displaying a current–bias curve under chopped light irradiation. Reprinted with permission from ref (80). Copyright 2022, American Chemical Society.

Au nanoparticles are commonly utilized as cocatalysts to enhance the surface photocatalytic activity of semiconductors, exploiting the surface plasmon resonance (SPR) effect.^74−76^ The properties of Au cocatalysts, including their size, shape, and distribution, significantly influence their photocatalytic performance.^77^ To gain insights into the specific sites of photocatalytic reactions and the SPR effect in Au-modified TiO_2_ systems, super-resolution mapping techniques employing fluorescent probe molecules have been employed.^78^ These studies have revealed that catalytic reactions induced by the fluorescent probes occur on the entire TiO_2_ surface (Figure 13a), whereas individual Au nanoparticles in Au-modified TiO_2_ systems exhibit limited active centers (Figure 13b). The spatial correlation observed between the reactive sites and the positions of Au nanoparticles provides further evidence of plasmon-induced photochemical reactions. In another investigation involving Au-modified CdS nanorods, a unique heterostructure was synthesized by incorporating Au tips onto CdS nanorods.^79^ The primary objective of this study was to identify the specific locations where holes and electrons exhibit enhanced reactivity during photocatalytic processes. Under excitation from a 405 nm laser, it was observed that the hole-induced active sites were distributed throughout the entire length of the CdS nanorods, while the electron-induced active sites were primarily concentrated at the ends of the nanorods. These findings shed light on the spatial distribution of charge carrier reactivity and offer valuable insights for the rational design of cocatalysts.

In situ conductive AFM can also provide direct insights into the photovoltage distribution and charge transfer processes at the interface of the ionomer/catalyst–semiconductor mimic structure, shedding light on the mechanisms of photo(electro)catalysis.^80^ A novel ionomer/catalyst–semiconductor structure is developed to mimic the realistic catalytic reaction conditions of photocatalysts in electrolytes. To obtain spatially resolved pseudo in situ photovoltage information, tunneling AFM (TUNA) current-sample bias curves are measured using the conductive AFM while illuminating the particles during scanning. The photovoltage distribution can be derived on the {101} facets of the BiVO_4_ photocatalyst particles; both the CoO_*x*_ and Pt nanoparticles exhibit positive photovoltage values. However, the photovoltage of the CoO_*x*_ nanoparticles is significantly larger than that of the Pt nanoparticles. In contrast, no measurable photovoltage is observed on the {001} facet, which is considered to be a photogenerated electron-favorable facet. Further analysis reveals that the CoO_*x*_-BiVO_4_–H_2_O interface exhibits rectifying behavior, deviating significantly from ohmic behavior. This indicates the selective collection of holes at this interface, emphasizing its role in facilitating hole transfer and separation in the photocatalytic system.

## 4 Conclusion

Functional imaging techniques, as opposed to typical structural imaging methods, have proven to be valuable tools for investigating various aspects of semiconductor materials at the single-particle level, including heterogeneous catalytic activity, interface defect states, and surface potentials. These techniques enable the construction of a comprehensive local structural profile of semiconductor materials, which in turn guides the rational design of high-performance catalysts. However, there are several outstanding challenges that need to be addressed to further advance the field.

Current functional imaging techniques primarily establish direct correlations between performance and initial structural information (e.g., size or shape). To overcome this limitation, there is a need to develop multimodal and multiscale functional imaging approaches that can correlate diverse structural and functional information.^81−83^ By incorporating different imaging modalities and scales, researchers can gain a more holistic understanding of the intricate relationship between catalyst structure and activity. Additionally, the application of advanced techniques such as deep learning can expedite the analysis of imaging data, allowing for more efficient and accurate interpretation.

The understanding of failure mechanisms in semiconductor catalysts at the single-particle level is still limited. Most functional imaging investigations of catalyst particles are conducted for relatively short durations, typically less than 1 h. To address this limitation, it is crucial to develop long-term imaging methods that can capture the performance–structure degradation mechanisms over extended periods. Furthermore, there is a strong demand for emerging functional imaging tools, exemplified by optical fiber-coupled scanning electrochemical microscopy^84^ and super-resolution electrochemiluminescence microscopy.^85,86^ These tools hold immense potential in revealing critical perspectives on the stability and durability of photocatalysts, thereby playing a pivotal role in advancing the design of more resilient materials.

Achieving a high signal-to-noise ratio is essential for single-particle characterization methods. For example, while high-performance EMCCD cameras enable single-photon detection,^85^ the current electrical current measurements in photocurrent microscopy often operate at the nanoampere level due to the background current of large-sized ITO electrodes used in functional imaging tests. To improve the accuracy of current signal detection, the development of microelectrode-integrated imaging techniques shows promise.^87^ Such techniques could potentially enhance the sensitivity and enable measurements at the picoampere level, providing more precise information on the charge carrier dynamics and reactivity of individual catalyst particles.

Addressing these limitations and pushing the boundaries of functional imaging techniques will undoubtedly advance our understanding of semiconductor catalysts and pave the way for the design of next generation low-cost high-performance materials with enhanced photocatalytic activities and stability.

## Glossary

functional imaging: imaging techniques that offer crucial insights into the performance of materials beyond their structural properties
single-molecule fluorescence microscopy: imaging techniques enable the visualization and study of individual molecules by detecting the fluorescence emitted when these molecules are excited
photoluminescence: the re-emission of light with a longer wavelength observed under light excitation
surface photovoltage: voltage difference generated at a material’s surface under light excitation, revealing its electronic characteristics and surface states.
photocurrent: electric current generated in a material as a direct result of the absorption of photons
